# Influence of Cooking Methods on Glucosinolates and Isothiocyanates Content in Novel Cruciferous Foods

**DOI:** 10.3390/foods8070257

**Published:** 2019-07-12

**Authors:** Nieves Baenas, Javier Marhuenda, Cristina García-Viguera, Pilar Zafrilla, Diego A. Moreno

**Affiliations:** 1 Institute of Nutritional Medicine, University Medical Center Schleswig-Holstein, Campus Lübeck, Ratzeburger Allee 160, 23538 Lübeck, Germany; 2 Faculty of Health Sciences, Department of Pharmacy, Universidad Católica San Antonio de Murcia (UCAM), Campus de los Jerónimos, Guadalupe, E-30107 Murcia, Spain; 3 Phytochemistry and Healthy Foods Laboratory, Research Group on Quality, Safety and Bioactivity of Plant Foods, Department of Food Sciences and Technology, CEBAS-CSIC, Campus de Espinardo-25, E-30100 Murcia, Spain

**Keywords:** *Brassica*, stir-frying, steaming, boiling, HPLC-DAD-ESI-MS/MS, UHPLC-QqQ-MS/MS, sulforaphane, iberin

## Abstract

*Brassica* vegetables are of great interest due to their antioxidant and anti-inflammatory activity, being responsible for the glucosinolates (GLS) and their hydroxylated derivatives, the isothiocyanates (ITC). Nevertheless, these compounds are quite unstable when these vegetables are cooked. In order to study this fact, the influence of several common domestic cooking practices on the degradation of GLS and ITC in two novel *Brassica* spp.: broccolini (*Brassica oleracea* var *italica* Group x *alboglabra* Group) and kale (*Brassica oleracea* var. *sabellica* L.) was determined. On one hand, results showed that both varieties were rich in health-promoter compounds, broccolini being a good source of glucoraphanin and sulforaphane (≈79 and 2.5 mg 100 g^−1^ fresh weight (F.W.), respectively), and kale rich in glucoiberin and iberin (≈12 and 0.8 mg 100 g^−1^ F.W., respectively). On the other hand, regarding cooking treatments, stir-frying and steaming were suitable techniques to preserve GLS and ITC (≥50% of the uncooked samples), while boiling was deleterious for the retention of these bioactive compounds (20–40% of the uncooked samples). Accordingly, the appropriate cooking method should be considered an important factor to preserve the health-promoting effects in these trending *Brassica*.

## 1. Introduction

There is epidemiological evidence of the benefit of consuming cruciferous foods on the reduction of risk of major chronic and degenerative diseases, such as cancer and cardiovascular and obesity-related metabolic disorders, due to their phytochemical composition [1,2]. 

Glucosinolates (GLS) are characteristic bioactive compounds of *Brassica* vegetables and can be classified as aliphatic, aromatic, or indoles based on their precursor amino acid and the types of modification to the variable R group [3]. In intact plant tissues, GLS are stored physically separated from compartments containing myrosinase enzymes (thioglucohydrolase, E.C. number 3.2.1.147), which are responsible for the hydrolysis of GLS to their respective bioactive isothiocyanates (ITC) and indoles. 

There is growing evidence that ITC exert antioxidant, anti-inflammatory and multi-faceted anticancer activities in cells, through the in vivo inhibition of inflammation pathways and activation of detoxification enzymes [2,4]. Therefore, the highest benefit of cruciferous foods occurs when they are consumed raw, avoiding the degradation of the enzyme myrosinase by cooking or processing. The hydrolysis of GLS to ITC and indoles is crucial for the health-promoting activities related to cruciferous consumption, and is produced after the loss of the cellular integrity because of tissue disruption, by crushing or chewing, or by the action of the gut microbiota [5,6]. 

However, the formation of ITC could be dramatically decreased due to different processing techniques, as the excessive heat exposure that may increase the degradation of GLS by myrosinase, and, consequently, significantly altering the ITC and indole levels [7,8]. In this respect, during the past decade, the effects on GLS contents of domestic culinary methods, such as steaming, microwaving, boiling and stir-frying, have been widely studied, mainly in broccoli, Brussels sprouts, cauliflower or cabbage [7,8,9,10]. These processing methods induce significant changes in the biochemical composition of crucifers, temperature and time being two crucial factors to be considered on the degradation rate of bioactive compounds while cooking. Other factors that may affect the stability of GLS are the endogenous myrosinase activity and the food matrix [11].

In recent years, the consumption of trending *Brassica* vegetables such as broccolini, a hybrid between conventional broccoli (*Brassica oleracea* var. *italica*) and Chinese kale (*B. oleracea* var. *alboglabra*), and kale (*Brassica oleracea* var. *sabellica* L.), has become a popular alternative to other members of this family, such as broccoli or cauliflower. Such vegetables also include health-promoting compounds, are softer, have a more acceptable flavor and taste, and have similar nutritional values [12,13]. Only a few publications have shown the GLS profile of broccolini [14,15,16], while kale, has been more extensively analyzed and shown to have a significant difference in its individual GLS profile, depending on the cultivar and geographical origin [17,18]. 

In this work, the total and individual GLS content and the presence of the characteristic ITCs (sulforaphane and iberin present in broccolini and kale, respectively), before and after being cooked by different methods (stir-frying, steaming, and boiling), have been studied in broccolini and kale as novel *Brassica* varieties with potential therapeutic effects.

## 2. Materials and Methods 

### 2.1. Plant Materials

Broccolini (Bimi^®^ *Brassica oleracea* var. *italica* x var. *alboglabra*) and kale (*Brassica oleracea* var. *sabellica* L.) were purchased from the local market in Murcia, Spain, and transported under refrigeration conditions directly to the laboratory. Then, vegetables were cut into uniform pieces (≈3 cm diameter and ≈10 cm stalk for broccolini samples, and strips ≈3–4 cm in width for kale without stem), mixed and sorted into 200 g samples to perform the different cooking methods: steaming, stir-frying and boiling (always in triplicate). Cooking conditions were determined by the nutritionists of our group based on traditional gastronomy. Additionally, an informal tasting panel (three trained people) assessed the final processed food in terms of sensorial features [19,20]. All the samples were cooked for 15 min, regardless of the cooking method, in order to make their effects comparable. Water (850 mL) was heated at 100 °C in a stainless-steel cooking pot, without pressure, and vegetables were added when water started to boil. For steaming, distilled water (500 mL) was added to a stainless-steel steamer, which was covered with a lid until reaching 98 °C ± 2°C; then the vegetables were introduced, with the temperature maintained during the whole process. Finally, 15 mL of extra virgin olive oil was preheated to 120 °C in a sauce pan, for stir-frying, and then samples were added [20]. Each process was performed three times for the three cooking methods. After, samples were separately collected, drained, cooled on ice, flash frozen in liquid nitrogen, and stored at −80 °C prior to analysis.

### 2.2. Extraction and Determination of Glucosinolates (GLS)

The extraction, determination and quantification of glucosinolates were carried out according to Baenas et al. (2014) [21]. Briefly, freeze-dried samples (100 mg) were extracted with 1 mL methanol 70% for 30 min at 70 °C, with shaking every 5 min using a vortex stirrer, and centrifuged (17500× *g*, 15 min, 4 °C). Supernatants were collected, and methanol was completely removed using a rotary evaporator. After suspending the samples in 1 mL MilliQ-H_2_O, GLS were first identified following their MS^2^ [M−H]^−^ fragmentations in Reverse Phase High Performance Liquid Cromatography (HPLC) equipped with diode array dectector (DAD) coupled to mass spectrometer (MS) using Electro spray ionization (ESI) in negative mode for the analyses (HPLC-DAD-ESI-MS^n^ Agilent Technologies, Waldbronn, Germany). Then, GLS were quantified using an HPLC-DAD 1260 Infinity Series (Agilent Technologies, Waldbronn, Germany) method in accordance with the order of elution already described for their identification and UV-Vis characteristic spectra. Water:trifluoroacetic acid (optima LC/MS from Fisher Scientific Co., Fair Lawn, NJ, US) (99.9:0.1, *v*/*v*) and acetonitrile (LC-MS-grade quality from HiPerSolv Chromanorm, BDH Prolabo, Leuven, Belgium) were used as mobile phases A and B, respectively, with a flow rate of 1 mL min^−1^. The linear gradient started with 1% of solvent B, reaching 17% solvent B at 15 min up to 17 min, 25% at 22 min, 35% at 30 min, and 50% at 35 min, which was maintained up to 45 min. The separation of intact GLS was carried out on a Luna C18 100A column (250 × 4.6 mm, 5 μm particle size; Phenomenex, Macclesfield, UK). Chromatograms were recorded at 227 nm, using sinigrin and glucobrassicin (Phytoplan, Germany), as external standards of aliphatic and indole GLS, respectively.

### 2.3. Extraction and Determination of Isothiocyanates (ITC)

The determination and quantification of ITC was carried out as defined by Baenas et al. (2017) [22]. In short, freeze-dried samples (50 mg) were extracted with 1.6 mL of MilliQ-H_2_O for 24 h at room temperature. Then, samples were centrifuged (17500× *g*, 5 min) and supernatants were collected for ITC measurements. The sulforaphane (SFN) and Iberin (IB) were analyzed following their Multiple Reaction Monitoring (MRM ) transitions by a UHPLC-QqQ-MS/MS method (Agilent Technologies, Waldbronn, Germany), according to Rodriguez-Hernández et al. (2013) [23]. The mobile phases employed were solvent A (H_2_O/ammonium acetate 13 mM (pH 4) (with acetic acid); 99.99:0.01, *v*/*v*) and solvent B (acetonitrile/acetic acid; 99.99:0.1, *v*/*v*). The flow rate was 0.3 mL min^−1^ using the following linear gradient: 60% of solvent B up to 0.7 min, 73% at 0.71 min up to 1 min, 100% at 1.01 min up to 3.5 min, and 60% at 3.51 min. Chromatographic separation was carried out on a ZORBAX Eclipse Plus C18 column (2.1 × 50 mm, 1.8 μm) (Agilent Technologies, Waldrom, Germany).

### 2.4. Statistical Analysis

Regarding statistical methods, all assays were conducted in triplicate. Data were processed using the SPSS 15.0 software package (LEAD Technologies, Inc., Chicago, IL, USA). We carried out a multifactorial analysis of variance (ANOVA) and Tukey’s multiple range test to determine significant differences at *p*-values <0.05.

## 3. Results and Discussion

### 3.1. Glucosinolates Content of Vegetables: Effects of Cooking Methods

In this work, broccolini and kale were selected as novel little-studied food matrices. These vegetables showed distinct profiles and great differences in GLS content (Figure S1). The GLS profile of these vegetables, along with their retention times and molecular ions [M–H]^−^ (m/z) are shown in Table 1. 

Fresh broccolini presented a total amount of 178 ± 3.4 mg GLS 100 g^−1^, the predominant GLS being the aliphatic glucoraphanin (GRA) (44% of the total) and the indole glucobrassicin (GB) (40%), followed by the indole neoglucobrassicin (NEO) (24%), the aliphatic progoitrin (PRO) (18%), the indoles 4-methoxyglucobrassicin (MGB) (8.5%) and 4-hydroxiglucobrassicin (HGB) (5%), and trace amounts (below the Limit of Quantitation (LOQ) of gluconapin, glucosinalbin, glucobrassicanapin and gluconasturtin (Table 2). These results are similar to those found in broccolini vegetable [15] and broccolini seeds [16].

On the other hand, kale showed lower content of GLS (54.5 ± 7.3 mg 100 g^−1^), sinigrin (SIN) being the main aliphatic GLS in uncooked samples (68 % of the total), followed by glucoiberin (GIB) (21%), and the indoles GB (4.4%), MGB (3.5%) and HGB (2.4%) (Table 2). Similar GLS profiles and contents were previously found in kale samples, the aliphatic SIN and GIB being the predominant GLS [24,25,26]. 

Both cruciferous vegetables presented higher contents of aliphatic than indole GLS (Figure S1), according to previous reports of *B. oleraceae* and *B. rapa* varieties [24,27], the presence of these aliphatic GLS being related to potent anti-cancer effects in cells. This is due to the bioactivity of their hydrolysis compounds (isothiocyanates), such as iberin, sulforaphane and allyl isothiocyanates [28]. 

Total GLS content in broccolini and kale, after cooking, showed differences due to the method used, with boiling the most unfavorable method for the degradation of these bioactive compounds (>85% loss in both varieties). When comparing steaming and stir-frying methods, in broccolini samples no significant differences were found, with the GLS loss around 50% compared to the uncooked samples. Nevertheless, the stir-frying treatment preserved 50% of the total GLS in kale samples, while steaming preserved just 35% (Table 2). These results are in agreement with previous publications using broccoli samples [29,30]; in contrast, some authors reported almost no changes in GLS concentration after steaming of broccoli [19,31]. According to previous reports, the vegetable matrix is a determining factor in the degradation rate of bioactive compounds during processing, as well as other plant-intrinsic factors, such as activity of myrosinase and the presence of specifier proteins, and extrinsic postharvest factors (e.g., domestic preparation or mastication) [11]. 

Uncooked broccolini presented large quantities of glucoraphanin (78 mg 100 g^−1^ F.W.) (Table 2), similar to those found in broccoli, but higher than what has been previously described for other *Brassica oleracea* vegetables, such as cauliflower and Brussels sprouts [32,33,34]; more than 70% of this GLS was maintained after steaming and stir-frying (57 mg 100 g^−1^ F.W.), according to Vallejo et al. (2002). This is of special interest as, so far, this is one of the most studied aliphatic GLS, due to the health-promoting properties of SFN, its derived isothiocyanate [35]. In addition, the indole GLS glucobrassicin accounted for almost 40% of the total GLS in broccolini. This is also remarkable as the hydrolysis of this compound toindole-3-carbinol, which undergoes self-condensation in the stomach to form 3,3′-diindoylmethane (DIM), provides anticarcinogenic activities [36].

In kale samples, the main GLS, sinigrin, was dramatically degraded after steaming or stir-frying (>80%), while glucoiberin was better preserved, rendering a 60% preservation after steaming and 80% after stir-frying (Table 2). Our results agree with previous reports that showed glucoiberin as the main aliphatic GLS in processed kale, as well as in a beverage made of apple juice with added freeze-dry or frozen kale [37] and after blanching, boiling or freezing kale [32].

Regarding indole GLS in kale, it is worth mentioning that after stir-frying, the contents of 4-hydroxiglucobrassicin, glucobrassicin and 4-methoxyglucobrassicin were statistically higher (two-fold) compared to fresh samples, while after steaming no statistically differences were found (Table 2). This fact could be supported by different mechanisms, such as a higher chemical extractability of GLS after moderate thermal treatments, resulting in a higher extraction of GLS in the laboratory [38], or due to a limited hydrolysis of GLS with stir-frying, as vegetables are only partially in contact with the sauce-pan. This was suggested by Verkerk et al. (2001), who found an increase in indole GLS (three–five-fold) after chopping broccoli and red cabbage [39].

In general, aliphatic GLS were better conserved after cooking methods compared to indole GLS, according to other authors [30,40], who showed indole GLS more sensitive to heat and to diffusion in cooking water while boiling. According to our results, steaming and stir-frying allowed the preservation of higher quantities of bioactive compounds, probably explained by the lack of water in direct contact with the vegetable, thus confirming previous work where the greater losses of these compounds were due to high temperatures in cooking water and leaching of compounds [19,34]. 

### 3.2. Isothiocyanate Content of Vegetables: Effects of Cooking Methods

**Figure 1:**
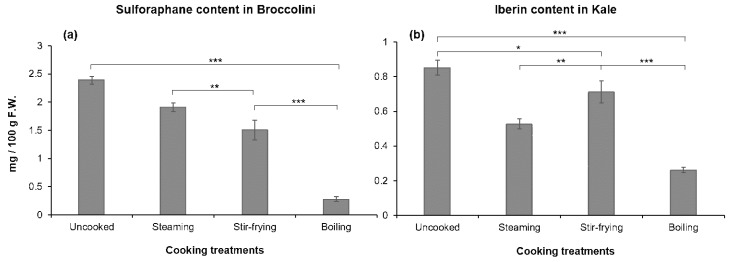
Isothiocyanate content in vegetables after cooking treatments: (**a**) sulforaphane content in broccolini samples; (**b**) iberin content in kale samples. F.W.: fresh weight. Mean values (*n* = 3) ± SD. Levels of statistically significant differences among treatments are the following: no differences at *p* > 0.05 (n.d.); significant at *p* < 0.05 (*); significant at *p* < 0.01 (**); significant at *p* < 0.001 (***).

Concerning ITC content, the concentration of sulforaphane (SFN) in broccolini samples and the content of iberin (IB) in kale samples were studied, according to the presence of their parental GLS, glucoraphanin and glucoiberin, respectively. In addition, both ITC were selected because of their documented relation to anti-inflammatory and anticancer activities in human cell lines [41,42]. Our results indicated a significant reduction of ITC contents after cooking, the highest being losses after boiling (Figure 1). The amount of SFN in raw samples of broccolini was 2.4 mg 100 g^−1^ F.W., decreasing after steaming (by 20%), stir-frying (by 36%), and boiling (by 88%) (Figure 1a). These results are interesting as they have not been described by other authors before, with Martinez-Hernandez et al. (2013) showing huge loses of SFN (>99%) in broccolini (kai lan-hybrid broccoli) after cooking, perhaps due to different processing treatments and analytical methods [29]. In addition, SFN contents were reported in broccoli florets after cooking by Jones et al. (2010), who showed loses of this ITC ranged from 20 to 50% after steaming, microwaving and boiling, these contents being lower than those shown in the present work after steaming and stir-frying [43]. Other authors have studied the effect of boiling on other *Brassica* spp., such as Brussels sprouts [44] or broccoli heads [45], where the presence of SFN was not found. This fact highlighted broccolini to be a variety that has to be more deeply investigated regarding the influence of processing on its composition and potential health effects [46,47].

In uncooked kale samples, the iberin content (0.8 mg 100 g^−1^ F.W.) was similar to that reported for cabbage [48] and higher than for turnip [49]. This ITC was better conserved when cooked under stir-frying conditions, showing a loss of only 17% of the total (Figure 1b). The values of this ITC in the cooking samples varied from 0.7 to 0.3 mg 100 g^−1^ F.W. This is, as far as we are aware, the first publication showing the effect of cooking methods on the iberin content in kale.

It is worth noting that sulforaphane, from broccolini, and iberin, from kale, were still present in the samples after being processed, so the enzyme myrosinase was still able to hydrolyze the GLS in the cooked samples. This loss of the ITCs during cooking could be explained by different mechanisms: (1) The enzyme myrosinase could be denatured during the high temperature treatments, resulting in a lower conversion of GLS to ITC during and after mastication [50]; (2) the loss of GLS at high temperatures or leached out into the boiling water would decrease the amount of ITC found in the processed vegetables [34]; and, (3) ITC could be volatilized while cooking [31]. It is also important to note the role of temperature in cooking processes, as mild heating (60–70 °C) selectively inactivates epithiospecifier proteins (ESP), while retaining myrosinase activity, avoiding the formation of nitrile products from GLS and increasing the formation of ITC [51]. Therefore, the multifactorial conditions affecting the ITC formation need further research to enhance the health benefits of *Brassica* consumption. According to recent research, cooked *Brassica* vegetables could also be consumed with an additional source of myrosinase, such as daikon radish, rocket or rape sprouts, promoting the hydrolysis of GLS to the bioactive ITC [52]. Finally, it is important to highlight that the gut microbiome has myrosinase-like activity, enhancing the formation of ITC after consumption of cooked *Brassica* [5]. 

## 4. Conclusions

Cooking broccolini and kale affected GLS and ITC concentrations, with individual GLS being directly affected according to the cooking method. Steaming and stir-frying treatments are generally better for preserving the total GLS content, compared to the boiling method. Broccolini is a good source of glucoraphanin and sulforaphane, with steaming being a better method for preserving those bioactive compounds. On the other hand, stir-frying is preferred when cooking kale, as these samples present higher contents of bioactive compounds than when cooked under the other conditions. An increased bioavailability of dietary ITC may be achieved by avoiding excessive cooking of vegetables, mainly boiling, as greater formation of ITC may be achieved with active plant myrosinase in raw *Brassica* foods.

## Figures and Tables

**Table 1:** List of glucosinolates detected in *Brassica* vegetables.

Glucosinolate	Semi-Systematic Name	Rt (min)	[M-H]^−^ (*m*/*z*)	Broccolini	Kale
Glucoiberin	3-methylsulfinylpropyl-gls	4.0	422	0 ^1^	+
Progoitrin	2-hydroxy-3-butenyl-gls	4.2	388	+	0
Glucoraphanin	4-methylsulfinylbutyl-gls	4.6	436	+	+
Sinigrin	2-propenyl-gls	5.7	358	0	+
Gluconapin	3-butenyl-gls	7.8	372	+	+
4-Hydroxyglucobrassicin	4-hydroxy-3-indolylmethyl-gls	11.0	463	+	+
Glucosinalbin	4-hydroxybenzyl-gls	13.6	424	+	0
Glucobrassicanapin	4-pentenyl-gls	17.2	386	0	+
Glucobrassicin	3-indolylmethyl-gls	20.0	447	+	+
Gluconasturtin	2-phenylethyl-gls	22.1	422	+	+
4-Methoxyglucobrassicin	4-methoxy-3-indolylmethyl-gls	23.5	477	+	+
Neoglucobrassicin	*N*-methoxy-3-indolylmethyl-gls	25.8	477	+	0

^1^ “0” indicates absence and “+” indicates presence of the individual glucosinolate.

**Table 2:** Individual aliphatic, indole and total glucosinolates (mg 100 g^−1^ F.W.) present in broccolini and kale in fresh and cooked samples.

**Glucosinolates**	**Broccolini**							
	**Fresh**		**Steaming**		**Stir-frying**		**Boiling**	
Progoitrin	31.76	±6.6 ^1^						
Glucoraphanin	78.74	±11.6 ^a^	58.37	±4.3 ^b^	56.27	±3.5 ^b^	16.86	±6.3 ^c^
4-Hydroxiglucobrassicin	9.61	±1.1 ^a^	4.68	±2.4 ^b^	3.33	±1.2 ^b^	1.30	±1.5 ^b^
Glucobrassicin	72.11	±4.5 ^a^	27.07	±6.4 ^b^	10.08	±1.5 ^c^	7.16	±1.9 ^c^
4-Methoxyglucobrassicin	15.10	±0.7 ^a^	5.69	±2.1 ^b^	4.17	±0.3 ^b^	1.68	±0.5 ^c^
Neoglucobrassicin	43.55	±3.3 ^a^	21.51	±4.5 ^b^	8.84	±0.7 ^c^	5.93	±1.3 ^c^
Aliphatic	110.49	±7.8 ^a^	58.37	±4.3 ^b^	56.27	±3.5 ^b^	16.86	±6.3 ^c^
Indolic	68.26	±5.1 ^a^	31.88	±9.0 ^b^	16.34	±1.8 ^c^	8.91	±3.3 ^c^
Total	178.76	±3.4 ^a^	90.24	±13.1 ^b^	72.60	±4.7 ^b^	25.77	±8.9 ^c^
**Glucosinolates**	**Kale**							
	**Fresh**		**Steaming**		**Stir-frying**		**Boiling**	
Glucoiberin	11.58	±1.3 ^a^	8.05	±0.1 ^b^	9.32	±0.6 ^a^	3.45	±0.1 ^b^
Sinigrin	37.27	±6.6 ^a^	4.09	±0.5 ^c^	6.60	±0.2 ^b^	2.09	±0.5 ^c^
4-Hydroxiglucobrassicin	1.34	±0.2 ^b^	1.51	±0.2 ^b^	2.81	±0.4 ^a^	0.46	±0.2 ^c^
Glucobrassicin	2.44	±0.3 ^b^	3.32	±0.2 ^b^	6.13	±0.9 ^a^	0.55	±0.1 ^c^
4-Methoxyglucobrassicin	1.90	±0.1 ^a^	2.14	±0.5 ^a^	2.85	±0.8 ^a^	0.57	±0.1 ^b^
Aliphatic	48.85	±7.8 ^a^	12.14	±0.6 ^c^	15.92	±0.8 ^b^	5.54	±0.6 ^d^
Indolic	5.68	±0.6 ^b^	6.96	±0.2 ^b^	11.79	±1.6 ^a^	1.58	±0.2 ^c^
Total	54.54	±7.3 ^a^	19.11	±0.3 ^c^	27.71	±0.9 ^b^	7.12	±0.7 ^d^

^1^ Mean values (*n* = 3) ± standard deviation (SD). Different lower-case letters indicate statistically significant differences among cooking treatments. Statistically significant at *p* < 0.05. F.W.: fresh weight.

## Supplementary Materials

The following are available online at https://www.mdpi.com/2304-8158/8/7/257/s1, Figure S1: Identification of glucosinolates in fresh *Brassica* samples following their MS^2^ [M−H]^−^ fragmentations in HPLC-DAD-ESI-MS^n^.

## References

[1] Royston K.J., Tollefsbol T.O. (2015). The Epigenetic Impact of Cruciferous Vegetables on Cancer Prevention. Curr. Pharmacol. Rep..

[2] Sita G., Hrelia P., Tarozzi A., Morroni F. (2016). Isothiocyanates Are Promising Compounds against Oxidative Stress, Neuroinflammation and Cell Death that May Benefit Neurodegeneration in Parkinson’s Disease. Int. J. Mol. Sci..

[3] Fahey J.W., Zalcmann A.T., Talalay P. (2001). The chemical diversity and distribution of glucosinolates and isothiocyanates among plants. Phytochemistry.

[4] Stefanson A., Bakovic M. (2014). Dietary Regulation of Keap1/Nrf2/ARE Pathway: Focus on Plant-Derived Compounds and Trace Minerals. Nutrients.

[5] Angelino D., Jeffery E. (2014). Glucosinolate hydrolysis and bioavailability of resulting isothiocyanates: Focus on glucoraphanin. J. Funct. Foods.

[6] Dosz E.B., Ku K.-M., Juvik J.A., Jeffery E.H. (2014). Total Myrosinase Activity Estimates in *Brassica* Vegetable Produce. J. Agric. Food Chem..

[7] Soares A., Carrascosa C., Raposo A. (2017). Influence of Different Cooking Methods on the Concentration of Glucosinolates and Vitamin C in Broccoli. Food Bioprocess. Technol..

[8] Tabart J., Pincemail J., Kevers C., Defraigne J.-O., Dommes J. (2018). Processing effects on antioxidant, glucosinolate, and sulforaphane contents in broccoli and red cabbage. Eur. Food Res. Technol..

[9] Garcia-Viguera C., Soler-Rivas C. (2017). Effect of Cooking on the Bioactive Compounds. Frontiers in Bioactive Compounds.

[10] Pellegrini N., Chiavaro E., Gardana C., Mazzeo T., Contino D., Gallo M., Riso P., Fogliano V., Porrini M. (2010). Effect of Different Cooking Methods on Color, Phytochemical Concentration, and Antioxidant Capacity of Raw and Frozen Brassica Vegetables. J. Agric. Food Chem..

[11] Oliviero T., Verkerk R., Dekker M. (2018). Isothiocyanates from *Brassica* Vegetables-Effects of Processing, Cooking, Mastication, and Digestion. Mol. Nutr. Food Res..

[12] Block E., Qian M.C., Fan X., Mahattanatawee K. (2011). Challenges and Artifact Concerns in Analysis of Volatile Sulfur Compounds. Volatile Sulfur Compounds in Food.

[13] Martínez-Hernández G.B., Artés-Hernández F., Gómez P.A., Artés F. (2013). Comparative behaviour between kailan-hybrid and conventional fresh-cut broccoli throughout shelf-life. LWT-Food Sci. Technol..

[14] Martínez-Hernández G.B., Gómez P.A., Artés F., Artés-Hernández F. (2015). Nutritional quality changes throughout shelf-life of fresh-cut kailan-hybrid and ‘Parthenon’ broccoli as affected by temperature and atmosphere composition. Food Sci. Technol. Int..

[15] Martínez-Hernández G.B., Artés-Hernández F., Gómez P.A., Artés F. (2013). Induced changes in bioactive compounds of kailan-hybrid broccoli after innovative processing and storage. J. Funct. Foods.

[16] Yang Y., Zhang X. (2012). Extraction, Identification and Comparison of Glucosinolates Profiles in the Seeds of Broccolini, Broccoli and Chinese Broccoli. Solvent Extr. Res. Dev. Jpn..

[17] Nilsson J., Olsson K., Engqvist G., Ekvall J., Olsson M., Nyman M., Akesson B. (2006). Variation in the content of glucosinolates, hydroxycinnamic acids, carotenoids, total antioxidant capacity and low-molecular-weight carbohydrates in *Brassica* vegetables. J. Sci. Food Agric..

[18] Giorgetti L., Giorgi G., Cherubini E., Gervasi P.G., Della Croce C.M., Longo V., Bellani L. (2018). Screening and identification of major phytochemical compounds in seeds, sprouts and leaves of Tuscan black kale *Brassica oleracea* (L.) ssp *acephala* (DC) var. *sabellica* L.. Nat. Prod. Res..

[19] Vallejo F., Tomás-Barberán B., García-Viguera C. (2002). Glucosinolates and vitamin C content in edible parts of broccoli florets after domestic cooking. Eur. Food Res. Technol..

[20] Moreno D.A., López-Berenguer C., García-Viguera C. (2007). Effects of Stir-Fry Cooking with Different Edible Oils on the Phytochemical Composition of Broccoli. J. Food Sci..

[21] Baenas N., García-Viguera C., Moreno D.A. (2014). Biotic Elicitors Effectively Increase the Glucosinolates Content in *Brassicaceae* Sprouts. J. Agric. Food Chem..

[22] Baenas N., Suárez-Martínez C., García-Viguera C., Moreno D.A. (2017). Bioavailability and new biomarkers of cruciferous sprouts consumption. Food Res. Int..

[23] Rodríguez-Hernández M.C., Medina S., Gil-Izquierdo A., Martínez-Ballesta C., Moreno D.A. (2013). Broccoli isothiocyanates content and in vitro availability according to variety and origin. Maced. J. Chem. Chem. Eng..

[24] Cartea M.E., Velasco P., Obregón S., Padilla G., de Haro A. (2008). Seasonal variation in glucosinolate content in *Brassica oleracea* crops grown in northwestern Spain. Phytochemistry.

[25] Korus A., Słupski J., Gębczyński P., Banaś A. (2014). Effect of preliminary processing and method of preservation on the content of glucosinolates in kale (*Brassica oleracea* L. var. *acephala*) leaves. LWT-Food Sci. Technol..

[26] Velasco P., Cartea M.E., González C., Vilar M., Ordás A. (2007). Factors Affecting the Glucosinolate Content of Kale (*Brassica oleracea acephala* Group). J. Agric. Food Chem..

[27] Florkiewicz A., Ciska E., Filipiak-Florkiewicz A., Topolska K. (2017). Comparison of Sous-vide methods and traditional hydrothermal treatment on GLS content in *Brassica* vegetables. Eur. Food Res. Technol..

[28] Gründemann C., Huber R. (2018). Chemoprevention with isothiocyanates—From bench to bedside. Cancer Lett..

[29] Martínez-Hernández G.B., Artés-Hernández F., Colares-Souza F., Gómez P.A., García-Gómez P., Artés F. (2013). Innovative Cooking Techniques for Improving the Overall Quality of a Kailan-Hybrid Broccoli. Food Bioprocess. Technol..

[30] Yuan G., Sun B., Yuan J., Wang Q. (2009). Effects of different cooking methods on health-promoting compounds of broccoli. J. Zhejiang Univ. Sci. B.

[31] Rungapamestry V., Duncan A.J., Fuller Z., Ratcliffe B. (2007). Effect of cooking brassica vegetables on the subsequent hydrolysis and metabolic fate of glucosinolates. Proc. Nutr. Soc..

[32] Cieślik E., Leszczyńska T., Filipiak-Florkiewicz A., Sikora E., Pisulewski P.M. (2007). Effects of some technological processes on glucosinolate contents in cruciferous vegetables. Food Chem..

[33] Sarvan I., Kramer E., Bouwmeester H., Dekker M., Verkerk R. (2017). Sulforaphane formation and bioaccessibility are more affected by steaming time than meal composition during in vitro digestion of broccoli. Food Chem..

[34] Song L., Thornalley P.J. (2007). Effect of storage, processing and cooking on glucosinolate content of Brassica vegetables. Food Chem. Toxicol..

[35] Russo M., Spagnuolo C., Russo G.L., Skalicka-Woźniak K., Daglia M., Sobarzo-Sánchez E., Nabavi S.F., Nabavi S.M. (2018). Nrf2 targeting by sulforaphane: A potential therapy for cancer treatment. Crit. Rev. Food Sci. Nutr..

[36] Jeffery E.H., Stewart K.E. (2004). Upregulation of Quinone Reductase by Glucosinolate Hydrolysis Products from Dietary Broccoli. Methods Enzymol..

[37] Biegańska-Marecik R., Radziejewska-Kubzdela E., Marecik R. (2017). Characterization of phenolics, glucosinolates and antioxidant activity of beverages based on apple juice with addition of frozen and freeze-dried curly kale leaves (*Brassica oleracea* L. var. *acephala* L.). Food Chem..

[38] Verkerk R., Schreiner M., Krumbein A., Ciska E., Holst B., Rowland I., De Schrijver R., Hansen M., Gerhäuser C., Mitchen R. (2009). Glucosinolates in Brassica vegetables: The influence of the food supply chain on intake, bioavailability and human health. Mol. Nutr. Food Res..

[39] Verkerk R., Dekker M., Jongen W.M. (2001). Post-harvest increase of indolyl glucosinolates in response to chopping and storage of Brassica vegetables. J. Sci. Food Agric..

[40] Palermo M., Pellegrini N., Fogliano V. (2014). The effect of cooking on the phytochemical content of vegetables. J. Sci. Food Agric..

[41] Jadhav U., Ezhilarasan R., Vaughn S.F., Berhow M.A., Mohanam S. (2007). Iberin induces cell cycle arrest and apoptosis in human neuroblastoma cells. Int. J. Mol. Med..

[42] Wagner A.E., Terschluesen A.M., Rimbach G. (2013). Health promoting effects of brassica-derived phytochemicals: From chemopreventive and anti-inflammatory activities to epigenetic regulation. Oxid. Med. Cell. Longev..

[43] Jones R.B., Frisina C.L., Winkler S., Imsic M., Tomkins R.B. (2010). Cooking method significantly effects glucosinolate content and sulforaphane production in broccoli florets. Food Chem..

[44] Ciska E., Drabińska N., Honke J., Narwojsz A. (2015). Boiled Brussels sprouts: A rich source of glucosinolates and the corresponding nitriles. J. Funct. Foods.

[45] Galgano F., Favati F., Caruso M., Pietrafesa A., Natella S. (2007). The Influence of Processing and Preservation on the Retention of Health-Promoting Compounds in Broccoli. J. Food Sci..

[46] Bayat Mokhtari R., Baluch N., Homayouni T.S., Morgatskaya E., Kumar S., Kazemi P., Herman Y. (2018). The role of Sulforaphane in cancer chemoprevention and health benefits: A mini-review. J. Cell Commun. Signal..

[47] Briones-Herrera A., Eugenio-Pérez D., Reyes-Ocampo J.G., Rivera-Mancía S., Pedraza-Chaverri J. (2018). New highlights on the health-improving effects of sulforaphane. Food Funct..

[48] Luang-In V., Deeseenthum S., Udomwong P., Saengha W., Gregori M. (2018). Formation of Sulforaphane and Iberin Products from Thai Cabbage Fermented by Myrosinase-Positive Bacteria. Molecules.

[49] Vieites-Outes C., Lopez-Hernandez J., Lage-Yusty M.A. (2009). Modification of glucosinolates in turnip greens (*Brassica rapa* subsp. *rapa* L.) subjected to culinary heat processes. CyTA J. Food.

[50] Nugrahedi P.Y., Verkerk R., Widianarko B., Dekker M. (2015). A Mechanistic Perspective on Process-Induced Changes in Glucosinolate Content in *Brassica* Vegetables: A Review. Crit. Rev. Food Sci. Nutr..

[51] Bricker G.V., Riedl K.M., Ralston R.A., Tober K.L., Oberyszyn T.M., Schwartz S.J. (2014). Isothiocyanate metabolism, distribution, and interconversion in mice following consumption of thermally processed broccoli sprouts or purified sulforaphane. Mol. Nutr. Food Res..

[52] Liang H., Wei Y., Li R., Cheng L., Yuan Q., Zheng F. (2018). Intensifying sulforaphane formation in broccoli sprouts by using other cruciferous sprouts additions. Food Sci. Biotechnol..

