# Supplementary material for: Influence of Cooking Methods on Glucosinolates and Isothiocyanates Content in Novel Cruciferous Foods
Source: Foods. 2019 Jul 12;8(7):257. doi: 10.3390/foods8070257 (PMC6679111; doi:10.3390/foods8070257)
Supplement: Supplementary file 1 [file foods-08-00257-s001.pdf]

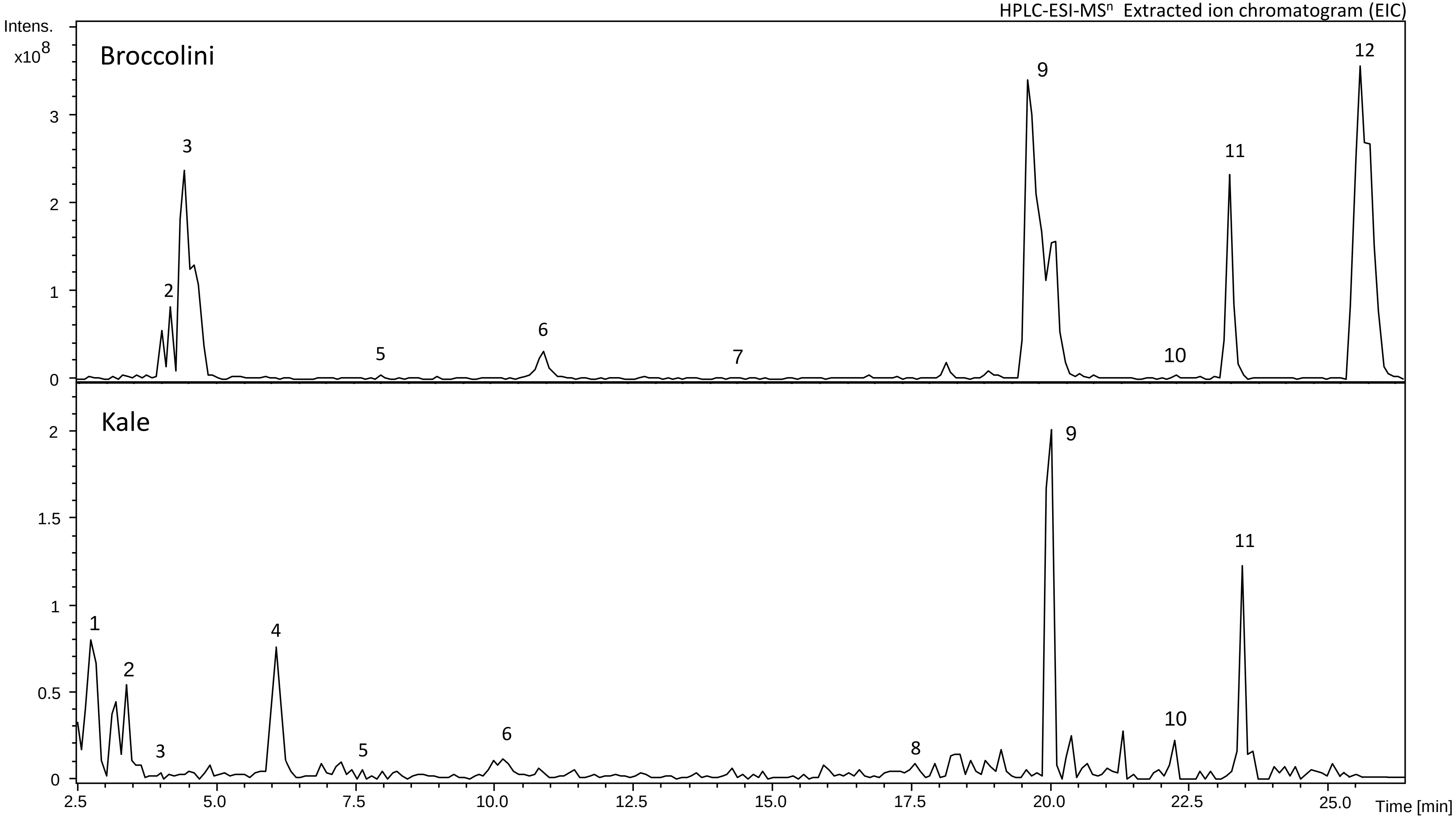

Identification of glucosinolates in fresh *Brassica* samples following their MS^2 $[M-H]^-$ fragmentations in HPLC-DAD-ESI-MS^n :

1. Glucoiberin (m/z 422)
2. Progoitrin (m/z 388)
3. Glucoraphanin (m/z 436)
4. Sinigrin (m/z 358)
5. Gluconapin (m/z 372)
6. 4-Hydroxyglucobrassicin (m/z 463)
7. Glucosinalbin (m/z 424)
8. Glucobrassicanapin (m/z 386)
9. Glucobrassicin (m/z 447)
10. Gluconasturtin (m/z 422)
11. 4-Methoxyglucobrassicin (m/z 477)
12. Neoglucobrassicin (m/z 477)
